# In Vivo Evaluation of a Nanoemulsion-Delivered Chromium(III)–Triazole Complex Against Fluconazole-Resistant *Candida albicans*

**DOI:** 10.3390/jof12060403

**Published:** 2026-06-02

**Authors:** Maria Valentina Bedoya-Florez, Ricardo A. Murcia-Galán, Martha Viviana Roa-Cordero, Sandra M. Leal-Pinto, Juan David Puerta-Arias, Yair Alvarez-Ricardo, John J. Hurtado, Tonny W. Naranjo

**Affiliations:** 1 Medical and Experimental Mycology Group CIB-UPB-UdeA-UDES, Medellín 050036, Colombia; 2 Research Group in Inorganic Chemistry, Catalysis, and Bioinorganic, Department of Chemistry, University of the Andes, Carrera 1 18A-12, Bogotá 111711, Colombia; ra.murcia@uniandes.edu.co (R.A.M.-G.); y.alvarezricardo@uniandes.edu.co (Y.A.-R.); jj.hurtado@uniandes.edu.co (J.J.H.); 3 Instituto de Investigación Masira, Facultad de Ciencias Médicas y de la Salud, Universidad de Santander, Bucaramanga 680002, Colombia; ma.roa@mail.udes.edu.co (M.V.R.-C.); sa.leal@mail.udes.edu.co (S.M.L.-P.); jua.puerta@mail.udes.edu.co (J.D.P.-A.); 4 School of Health Sciences, Universidad Pontificia Bolivariana, Medellín 050031, Colombia

**Keywords:** *Candida albicans*, chromium complexes, antifungal agents, nanoemulsion, invasive candidiasis, fluconazole resistance

## Abstract

*Candida albicans* remains one of the leading causes of invasive fungal infections and is recognized as a critical-priority pathogen by the World Health Organization. The increasing emergence of resistance to azole antifungals such as fluconazole highlights the need for alternative therapeutic strategies. In this study, we evaluated the antifungal potential of a chromium(III)–triazole coordination complex (CrL1) against *C. albicans*. In vitro susceptibility testing showed that CrL1 exhibited notable antifungal activity against the fluconazole-resistant strain with low cytotoxicity in murine macrophages. To facilitate aqueous dispersion and enable in vivo administration, CrL1 was incorporated into an oil-in-water nanoemulsion (NE-CrL1). The antifungal activity of NE-CrL1 was evaluated in a murine model of invasive candidiasis. In mice infected with a fluconazole-resistant *C. albicans* strain, treatment with NE-CrL1 reduced renal fungal burden and was associated with attenuation of histopathological alterations and changes in local inflammatory responses. Although the present study has limitations, including the absence of mechanistic assays and additional physicochemical characterization, these results suggest in vivo antifungal activity of NE-CrL1 and warrant further preclinical evaluation against drug-resistant *Candida* infections.

## 1. Introduction

Fungal infections affect more than one billion individuals worldwide each year. Of these, 150 million cases correspond to severe and life-threatening infections, resulting in approximately 1.7 million deaths annually. This trend is driven by several factors, including extended life expectancy, widespread use of immunosuppressive therapies, and the excessive administration of antimicrobial agents, all of which contribute to a heightened risk of developing invasive fungal infections (IFIs) [1].

Among the fungal pathogens implicated in IFIs, *Candida* species are among the most common, with invasive candidiasis being a life-threatening disease associated with high mortality. *Candida albicans* is the most prevalent, accounting for over 80% of yeast isolates from mucosal surfaces such as the skin, oral cavity, vaginal tract, and gastrointestinal tract in healthy individuals [2]. Although *C. albicans* is typically a commensal organism, it can shift to a pathogenic state when host immune defenses or the local microbiota are compromised, leading to either superficial infections or, in severe cases, invasive candidiasis (IC) [3]. Invasive candidiasis encompasses a wide spectrum of conditions, ranging from candidemia, a bloodstream infection, to deep-seated candidiasis. The latter arises from hematogenous dissemination involving vital organs such as the kidneys, liver, and brain, or by direct inoculation of *Candida* species into normally sterile sites such as the peritoneum, pleural space, or joints [4].

The therapeutic management of IC is complex and involves several antifungal agents depending on the patient’s immune status, the anatomical site of infection, and disease severity. Currently, there are three main classes of antifungal drugs: polyenes, azoles, and echinocandins, with the latter being considered first-line treatment for candidemia due to their efficacy, safety, and broad activity against *Candida* spp. Nevertheless, azoles such as fluconazole and voriconazole remain widely used, particularly as step-down or consolidation therapy, owing to their oral bioavailability, well-characterized pharmacokinetics, and established role in both prophylaxis and treatment of susceptible infections. Polyenes, such as amphotericin B, are typically reserved for patients who cannot tolerate or fail to respond to other antifungal agents, due to their higher toxicity [5]. However, all three antifungal classes present limitations, including high toxicity, drug–drug interactions, and limited antifungal spectrum. The rising incidence of antifungal resistance, particularly among fluconazole-resistant *Candida*, complicates clinical management and highlights the urgent need for novel therapeutic approaches [6].

In this context, the development of metal-based coordination complexes has emerged as a promising frontier in the search for novel antimicrobial agents [7,8]. However, relatively few studies have conducted comprehensive in vitro or in vivo evaluations of metal-containing antifungal compounds [9,10,11]. Chohan et al. [10] reported the synthesis of a series of cobalt(II), copper(II), nickel(II), and zinc(II) coordination complexes with demonstrated antifungal activity. Nevertheless, several of these complexes exhibited high cytotoxicity, limiting their therapeutic applicability. More recently, Murcia-Galán et al. [11] synthesized and characterized four novel compounds based on Co(II) complexes containing a benzotriazole-derived ligand, which showed notable in vitro inhibitory effect against fluconazole-resistant *Candida* isolates. Similarly, the same authors reported three chromium(III) coordination complexes (CrL1, CrL2 and CrL3), each containing a distinct triazole-based ligand, which showed significant antifungal effects against fluconazole-resistant *Candida* strains, along with lower cytotoxicity, underscoring their potential for further preclinical studies [9].

Chromium(III) coordination complexes have garnered attention due to their ability to generate reactive oxygen species (ROS), induce ROS-mediated apoptosis, and cause DNA damage, which may contribute to their antifungal activity. Additionally, Cr(III) exhibits high thermodynamic and kinetic stability, forming octahedral complexes capable of interacting with microbial biomolecules such as DNA and proteins, thereby impairing metabolic function and promoting membrane disruption. Although their activity has been more extensively characterized in antibacterial models, several Cr(III) complexes have demonstrated promising antifungal effects, including against *C. albicans*, highlighting their potential as novel therapeutic agents [12,13].

Based on this background, the present study aimed to evaluate the antifungal efficacy of a chromium-based coordination complex with a triazole ligand (CrL1) in a murine model of invasive candidiasis caused by *C. albicans*. Specifically, we assessed its effects on fungal burden, tissue damage, and host inflammatory responses to explore its potential as an alternative antifungal strategy against drug-resistant *Candida* infections.

## 2. Materials and Methods

### 2.1. Chromium Coordination Complexes and Reference Drugs

Three chromium(III) coordination complexes with triazole ligands (CrL1, CrL2, CrL3) previously synthesized by Murcia et al. were used in this study [9]. Their chemical structures are shown in Figure S1. Chromium chloride hexahydrate salt (CrCl_3_·6H_2_O) was obtained from Alfa Aesar (Ward Hill, MA, USA). Fluconazole and itraconazole, both from Sigma-Aldrich (St. Louis, MO, USA) were used as reference antifungal drugs for in vitro susceptibility testing. Diflucan^®^ (fluconazole, 2 mg/mL; Pfizer, New York, NY, USA) was used as the reference antifungal drug for in vivo treatment. For in vitro assays, the tested compounds were solubilized in dimethyl sulfoxide (DMSO).

### 2.2. Fungal Strains

*C. albicans* ATCC 90028 was obtained from the American Type Culture Collection (ATCC). The fluconazole-resistant *C. albicans* CAP F-13 strain used in this study is a clinical isolate deposited in the strain collection of Medical and Experimental Mycology Group at the Corporación para Investigaciones Biológicas (CIB, Medellín, Colombia). Both strains were maintained on Sabouraud Dextrose Agar (BD, Sparks, MD, USA), supplemented with 100 μg/mL streptomycin and 100 U/mL penicillin (Gibco, Thermo Fisher Scientific, Waltham, MA, USA) at 37 °C. Prior to each assay, fresh cultures were prepared, and cell suspensions were adjusted to the required cell concentration.

### 2.3. Minimal Inhibitory Concentration (MIC) Determination

The antifungal activity of coordination compounds, chromium salt, and triazole ligands was assessed using the broth microdilution method, in accordance with Clinical and Laboratory Standards Institute (CLSI) document M27-A4 [14]. An inoculum of 5 × 10^2^–2.5 × 10^3^ CFU/mL was exposed to serial dilutions of the test compounds (0.97–1000 µg/mL) in RPMI medium (Gibco, Thermo Fisher Scientific, Waltham, MA, USA). Microplates (96-well) were incubated at 35 °C for 24 h, after which the minimum inhibitory concentration (MIC) was visually assessed. To further assess fungicidal activity, subcultures from wells at the MIC and higher concentrations were plated on Sabouraud Dextrose Agar and incubated for 24 h at 35 °C. The minimum fungicidal concentration (MFC) was defined as the lowest concentration producing no visible growth. Fluconazole and itraconazole were included as reference antifungal drugs.

### 2.4. In Vitro Cytotoxicity Assay

Macrophages J774A.1 (ATCC TIB-67) were provided by the Cellular and Functional Biology and Biomolecular Engineering Group of the Universidad Antonio Nariño, Colombia. The cells were cultured in Dulbecco’s Modified Eagle Medium (D-MEM, Gibco, USA) supplemented with 10% heat-inactivated fetal bovine serum (Gibco, USA) and 1% penicillin/streptomycin solution. For cytotoxicity testing, a protocol previously used by our group was followed [9]. Briefly, J774A.1 cells (1 × 10^5^ cells/mL) were seeded into 96-well flat-bottom plates and incubated at 37 °C in a humidified 5% CO_2_ atmosphere for 24 h until a monolayer was formed. Cells were then treated with test compounds at concentrations ranging from 11.1 to 300 µg/mL and incubated under identical conditions. Cell viability was assessed using the MTT assay (Sigma-Aldrich, USA), according to the manufacturer’s instructions. Absorbance was measured at 590 nm using an iMark™ microplate reader (Bio-Rad, Madrid, Spain). Half-maximal cytotoxic concentration (CC_50_) values were calculated via sigmoidal regression using Xlfit™ 4 software (IDBS, Guilford, UK).

### 2.5. Preparation and Preliminary Characterization of Nanoemulsion

The complex showing the highest anti-*Candida* activity was encapsulated to improve its aqueous dispersion. For this purpose, an oil-in-water nanoemulsion was prepared using high-energy ultrasonic emulsification, adapted from Sugumar et al. [15], with castor oil (14% *v*/*v*) as the oil phase and Tween 80 (26% *v*/*v*) as the surfactant. The complex CrL1 was first dissolved in castor oil at temperatures below 50 °C for 20 min, followed by the gradual addition of Tween 80 under constant stirring at 50 °C for another 20 min. Subsequently, Milli-Q water was added to reach a final volume of 20 mL. Ultrasonic homogenization (Ivymen Ultrasonic System) was performed for 10 min using 60-s pulses at 90% amplitude, resulting in the formation of a transparent nanoemulsion, hereafter referred to as NE-CrL1. An Unloaded NE was prepared using the same procedure but without CrL1. Colloidal characterization of the nanoemulsion was conducted by measuring the hydrodynamic diameter (*D*_h_), polydispersity index (PDI), and zeta potential (ZP) using dynamic light scattering (DLS) and electrophoretic light scattering (ELS) (Zetasizer ZS90, Malvern Panalytical, Malvern, UK). Nanoemulsion stability was monitored over a four-month storage period at 8 °C to assess potential changes in colloidal properties.

### 2.6. In Vivo Model of Invasive Candidiasis

Female BALB/c mice (8–10 weeks old) were obtained from the breeding colony at Corporación para Investigaciones Biológicas (CIB, Medellín, Colombia) and maintained under controlled conditions (21 ± 1 °C, 45–65% humidity, 12 h light/dark cycle) and with free access to sterile food and water. The animal model of systemic candidiasis was established based on protocols adapted from the National Institute of Allergy and Infectious Diseases (NIAID) [16]. Two strains of *C. albicans* with different fluconazole susceptibility profiles were used: fluconazole-susceptible (ATCC 90028) and fluconazole-resistant (CAP F-13). Intravenous inoculation of a yeast suspension, previously adjusted to 2.5 × 10^6^ cells/mL, was performed. Forty-five (*n* = 45) mice per strain were used and assigned by simple randomization to three experimental groups: infected-untreated, infected-FLZ, and infected-NE-CrL1. An additional uninfected-untreated control group was included (*n* = 10 per strain; 2 animals per time point). These animals were housed under identical conditions but were not inoculated. Antifungal treatment consisted of fluconazole (FLZ) 6 mg/kg/day or NE-CrL1 15 mg/kg/day, administered intraperitoneally once daily for three consecutive days, starting 2 h post-infection. The dose of NE-CrL1 was selected based on the in vitro MIC value of CrL1 against the fluconazole-resistant CAP F-13 strain (15.5 µg/mL), given that pharmacokinetic data for this novel compound are not yet established. Mice were monitored daily for clinical signs of illness, including weight loss, behavioral changes, and any signs of systemic infection.

### 2.7. Ethics Statement

The animal studies strictly followed the Colombian laws (Law 84/1989, Resolution No. 8430/1993) and the European Union Directive 2010/63/EU on animal care. The study was approved by the Ethics Committee of the University of Santander.

### 2.8. Colony Forming Units (CFU) Determination

Groups of mice were euthanized after 2, 24, 48, 72 and 96 h post-infection. Kidneys and brains were aseptically removed, weighed and homogenized using a GentleMACS Dissociator (Miltenyi Biotec, Bergisch Gladbach, Germany). Homogenization was performed in 1 mL of sterile phosphate-buffered saline (PBS) containing 100 U/mL penicillin, 100 µg/mL streptomycin, and protease inhibitor cocktail (Roche Applied Science, Mannheim, Germany). Tissue homogenates were diluted (1:1000) and 0.1 mL was plated on Sabouraud Dextrose Agar medium, followed by incubation at 37 °C. CFU counts were performed after 24 h and expressed as log_10_ CFU/g of tissue.

### 2.9. Histopathological Analysis

Kidney and brain samples were fixed in 4% formalin, paraffin-embedded, sectioned, and stained with hematoxylin and eosin (H&E). Histopathological evaluation was performed by an external certified veterinary pathology laboratory under blinded conditions, where the pathologist was unaware of the experimental group allocation. For each animal, two tissue sections per organ were analyzed. Semi-quantitative scoring was conducted using a standardized panel of twenty-four morphological parameters, graded on a severity scale from 1 (absence of lesion) to 6 (severe lesion). Two animals per experimental group per time point were included in the histological evaluation; therefore, findings are presented as descriptive observations. Representative microphotographs correspond to H&E-stained kidney sections at 96 h post-infection (final time point) and were selected based on lesion patterns consistent with the corresponding semi-quantitative scores. Higher magnification images (40×) correspond to the same fields identified at lower magnification (10×).

### 2.10. Determination of Immunomarkers

Kidney homogenates were centrifuged (500× *g*, 4 °C, 10 min), and supernatants were stored at −70 °C. Cytokine concentrations were quantified using a multiplex assay ProcartaPlex Mouse Th1/Th2 Cytokine Panel 11plex (ThermoFisher Scientific, Waltham, MA, USA) and the MAGPIX^®^ Luminex platform (EMD Millipore, Burlington, MA, USA). The molecules analyzed were granulocyte macrophage colony-stimulating factor (GM-CSF), IFN-γ, IL-1β, IL-2, IL-4, IL-5, IL-6, IL-12p70, IL-13, IL-18 and TNF-α. Cytokine levels were normalized to total protein concentration in each sample, which was determined using the Pierce™ BCA Protein Assay Kit (ThermoFisher Scientific, USA).

### 2.11. Statistical Analysis

Data analysis was performed using GraphPad Prism version 8.0 (GraphPad Software, Inc., San Diego, CA, USA). Normality was assessed with the Shapiro–Wilk test, and group comparisons were conducted using two-way ANOVA followed by Tukey’s post hoc test. Differences were considered statistically significant at values of *p* < 0.05.

## 3. Results

### 3.1. Antifungal Activity of CrL1 Against C. albicans and Cytotoxicity in Murine Macrophages

The antifungal activity of chromium(III) complexes (CrL1, CrL2, and CrL3), their corresponding triazole ligands (L1–L3), and the precursor salt CrCl_3_·6H_2_O was evaluated against planktonic cultures of *C. albicans* and cell cultures of murine macrophages (cell line J774). The free ligands did not exhibit detectable antifungal activity under the conditions tested (MIC > 1000 μg/mL), whereas their corresponding chromium complexes showed measurable antifungal activity (Table 1). Among them, CrL1 exhibited the lowest MIC values, particularly against the fluconazole-resistant CAP F-13 strain (MIC: 15.5 μg/mL). Consistently, CrL1 showed low cytotoxicity in murine macrophages (CC_50_ > 300 μg/mL), resulting in a selectivity index of 19.4. Notably, CrL1 displayed lower activity against the fluconazole-susceptible ATCC 90028 strain (MIC: 125 μg/mL, SI = 2.4), indicating a differential response depending on the strain evaluated.

In the case of nanoemulsion formulation, NE-CrL1 exhibited antifungal activity, with slightly higher MIC values compared to the free chromium complex and no apparent cytotoxicity in J774 macrophages. The Unloaded NE showed antifungal activity only at higher concentrations (MIC = 500 μg/mL), which may reflect the contribution of formulation components.

### 3.2. Preliminary Physicochemical Characterization of CrL1-Loaded Nanoemulsion

To enable in vivo evaluation of CrL1, the compound was incorporated into a nanoemulsion system. As an initial characterization, the hydrodynamic diameter (*D*_h_), polydispersity index (PDI) and zeta potential (ZP) of the nanoemulsion were evaluated throughout four months of storage at 8 °C. NE-CrL1 maintained a *D*_h_ below 86 nm and a consistent PDI of ~0.46 over time, indicating a relatively broad size distribution, compared to the Unloaded NE. Zeta potential values remained close to neutrality for NE-CrL1, with no significant variations over the evaluation period. Although the nanoemulsion remained within the measured size and PDI ranges over the storage period (Supplementary Table S1), further physicochemical characterization is required.

### 3.3. NE-CrL1 Reduced Renal Fungal Burden in Mice Infected with Fluconazole-Resistant C. albicans

**Figure 1:**
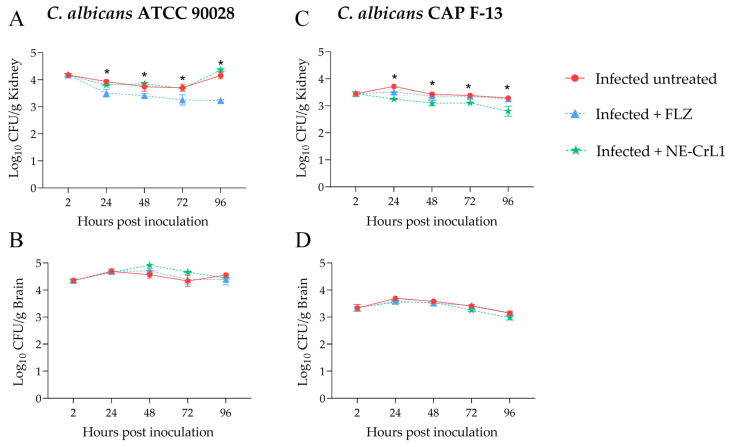
Fungal burden in kidney and brain tissue of infected mice. (**A**,**B**) Fluconazole-susceptible strain (ATCC 90028); (**C**,**D**) Fluconazole-resistant strain (CAP F-13). Colony-forming units (CFUs) were quantified per gram of tissue at multiple time points. * *p* < 0.05: vs. infected untreated mice.

Following formulation, the antifungal activity of NE-CrL1 was evaluated in a murine model of invasive candidiasis. Fungal burden was quantified in renal and brain tissues at multiple time points in mice infected with two distinct *C. albicans* strains: fluconazole-susceptible (ATCC 90028) and fluconazole-resistant (CAP F-13) (Figure 1). Infection and dissemination by both strains were confirmed by detectable colony-forming unit (CFU) counts in kidney and brain tissue as early as 24 h post-infection. Mice infected with ATCC 90028 exhibited higher fungal burden in both kidney and brain at the evaluated time points compared to those infected with CAP F-13 (Figure 1).

Consistent with the susceptibility profile, mice infected with *C. albicans* ATCC 90028 and treated with fluconazole (FLZ) showed a reduction in kidney fungal burden from 24 h post-infection onward. Conversely, treatment with NE-CrL1 did not result in a statistically significant change in fungal burden in this strain (Figure 1A).

In contrast, in mice infected with the fluconazole-resistant CAP F-13 strain, kidney fungal burden remained comparable between untreated and FLZ-treated groups. Treatment with NE-CrL1 was associated with a reduction in kidney fungal burden throughout the infection period. Notably, at 96 h post-infection, NE-CrL1-treated mice showed lower fungal burden (2.8 log_10_ CFU/g tissue) compared to untreated or FLZ-treated infected mice (3.3 log_10_ CFU/g tissue in both) (Figure 1C). No significant differences in brain fungal burden were observed across experimental groups for either strain (Figure 1B,D).

### 3.4. NE-CrL1 Was Associated with Attenuation of Histopathological Alterations in a Murine Model of Invasive Candidiasis

**Figure 2:**
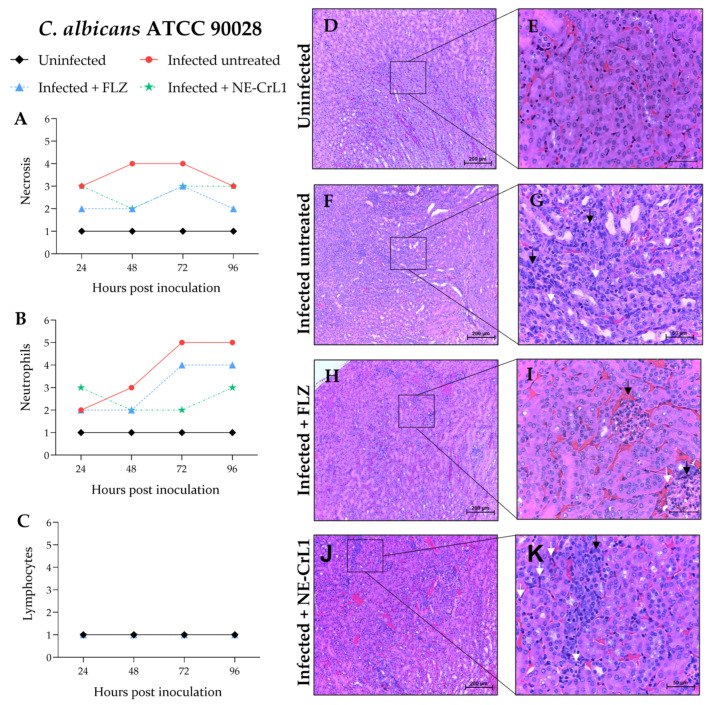
Semi-quantitative histopathological scoring of renal lesions in mice infected with *C. albicans* ATCC 90028 (fluconazole-susceptible strain). Kidney was evaluated for (**A**) necrosis, (**B**) neutrophil infiltration, and (**C**) lymphoid infiltration. (**D**–**K**). Each parameter was graded on a severity scale from 1 (no lesion) to 6 (severe lesion). Representative H&E-stained kidney sections at 96 h post-infection: uninfected (**D**,**E**), infected untreated (**F**,**G**), infected + FLZ (**H**,**I**), and infected + NE-CrL1 (**J**,**K**). Magnification 10× (**D**,**F**,**H**,**J**) and 40× (**E**,**G**,**I**,**K**); 40× images correspond to boxed areas at 10×. Uninfected control panels (**D**,**E**) are shared with Figure 3. Black arrows indicate neutrophilic infiltrates; white arrows indicate fungal structures.

**Figure 3:**
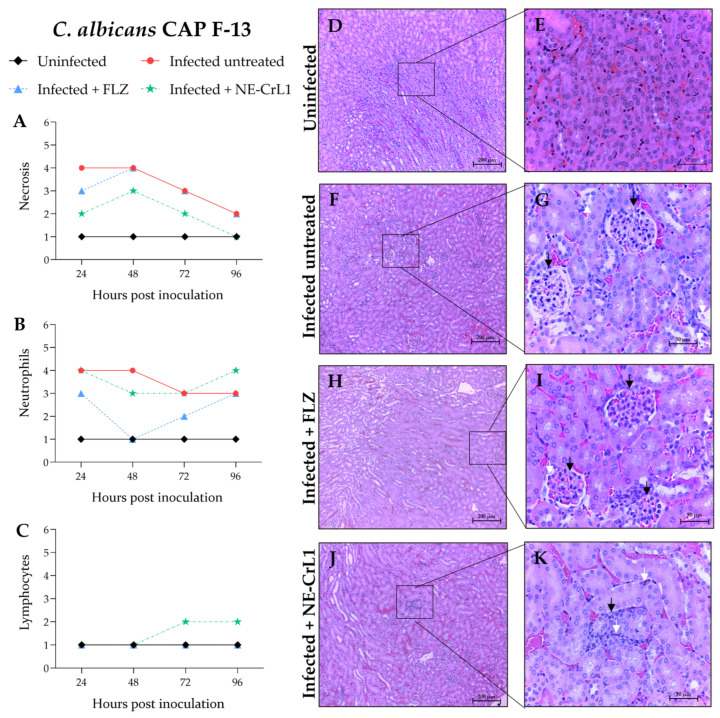
Semi-quantitative histopathological scoring of renal lesions in mice infected with *C. albicans* CAP F-13 (fluconazole-resistant strain). Kidney was evaluated for (**A**) necrosis, (**B**) neutrophil infiltration, and (**C**) lymphoid infiltration. (**D**–**K**). Each parameter was graded on a severity scale from 1 (no lesion) to 6 (severe lesion). Representative H&E-stained kidney sections at 96 h post-infection: uninfected (**D**,**E**), infected untreated (**F**,**G**), infected + FLZ (**H**,**I**), and infected + NE-CrL1 (**J**,**K**). Magnification 10× (**D**,**F**,**H**,**J**) and 40× (**E**,**G**,**I**,**K**); 40× images correspond to boxed areas at 10×. Uninfected control panels (**D**,**E**) are shared with Figure 2. Black arrows indicate neutrophilic infiltrates; white arrows indicate fungal structures.

To further assess tissue-level effects, kidney and brain tissues were analyzed histopathologically to evaluate alterations associated with invasive candidiasis and their evolution following NE-CrL1 treatment. In renal tissue, uninfected animals showed preserved parenchymal architecture with no evidence of inflammatory infiltrate (Figure 2D,E). In contrast, untreated infected mice with either strain showed progressive tissue alterations, including increased necrosis and neutrophilic infiltration from 24 h post-infection (Figure 2A,B and Figure 3A,B). The infiltrate showed a predominantly diffuse interstitial pattern in ATCC 90028-infected animals (Figure 2F,G) and a periglomerular pattern with glomerular congestion in CAP F-13-infected animals (Figure 3F,G). Structures morphologically compatible with fungal elements were also observed in infected untreated animals (Figure 2G and Figure 3G; indicated by white arrows).

In ATCC 90028-infected mice, treatment with NE-CrL1 was associated with lower neutrophilic infiltration scores and a trend toward reduced necrosis between 48 and 72 h post-infection (Figure 2A,B). Fluconazole treatment showed a more pronounced reduction in these parameters, consistent with the expected antifungal activity in this susceptible strain (Figure 2A,B,H,I). In CAP F-13-infected mice, fluconazole treatment did not result in consistent attenuation of tissue alterations (Figure 3A,B,H,I). In contrast, treatment with NE-CrL1 was associated with lower necrosis scores and increased lymphocytic infiltration at later time points (Figure 3A–C). Reduced inflammatory infiltrate and relatively better preserved tubular and glomerular architecture were observed compared to untreated and FLZ-treated infected groups (Figure 3J,K).

In brain tissue, NE-CrL1-treated mice showed lower necrosis scores compared to untreated infected mice. However, increased neutrophilic infiltration was also observed in some groups, which may reflect either a protective immune response or an inflammatory process (Figure S2).

### 3.5. Changes in Cytokine Responses Following NE-CrL1 Treatment in Murine Invasive Candidiasis

**Figure 4:**
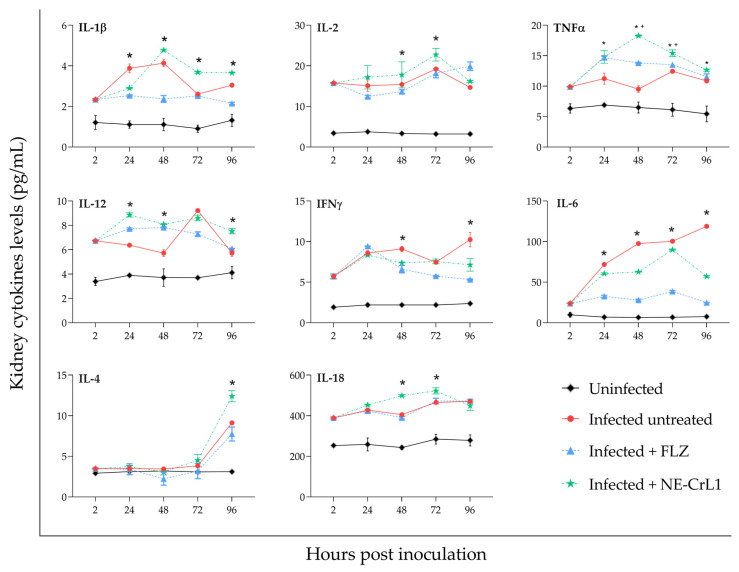
Cytokine levels in renal tissue of mice infected with fluconazole-susceptible *C. albicans* (ATCC 90028). Measurements were performed in kidney tissue from mice uninfected or untreated, FLZ-treated or NE-CrL1-treated infected mice at multiple time points. * *p* < 0.05: vs. infected untreated mice; + *p* < 0.05: vs. uninfected group.

**Figure 5:**
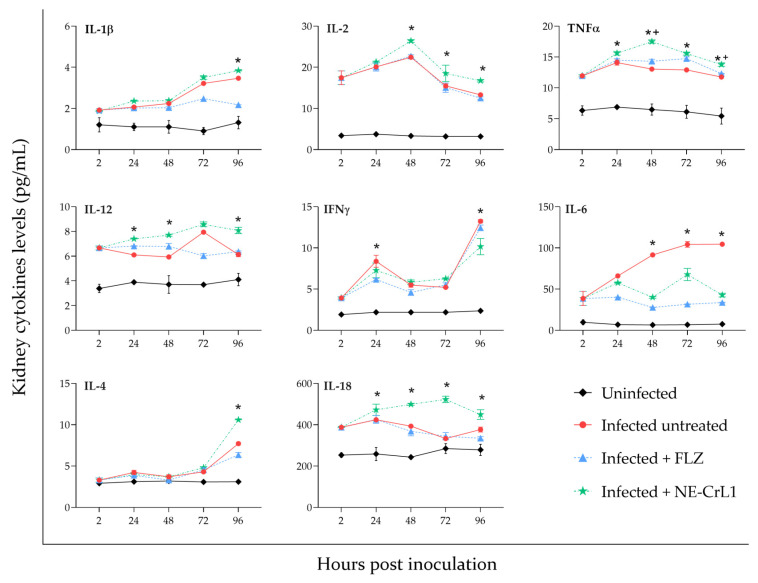
Cytokine levels in renal tissue of mice infected with fluconazole-resistant *C. albicans* (CAP F-13). Measurements were performed in kidney tissue from mice uninfected or untreated, FLZ-treated or NE-CrL1-treated infected mice at multiple time points. * *p* < 0.05: vs. infected untreated mice; + *p* < 0.05: vs. uninfected group.

The effect of NE-CrL1 treatment on the local inflammatory response was evaluated by measuring cytokine levels in renal tissue. Elevated levels of pro-inflammatory cytokines including IL-1β, IL-2, TNF-α, IL-12, IFN-γ, IL-6, and IL-18 were detected in infected mice with either strain compared to the uninfected control (Figure 4 and Figure 5). GM-CSF, IL-5, and IL-13 were also quantified but did not show consistent changes across experimental groups and are therefore not shown.

In mice infected with the ATCC 90028 strain, fluconazole treatment was associated with reduced IL-1β and IL-6 levels from 24 h post-infection, together with increased TNF-α and IL-12 (Figure 4). Conversely, in mice infected with the CAP F-13 strain, fluconazole treatment showed limited changes in cytokine levels compared to untreated infected mice.

In contrast, treatment with NE-CrL1 resulted in an increase in IL-1β, IL-2, IL-12, IL-18, and TNF-α in infected groups (with either strain) compared with infected-untreated or FLZ-treated infected groups (Figure 4 and Figure 5). Additionally, a late-stage increase in IL-4 levels was detected in the same groups of mice.

## 4. Discussion

In this study, treatment with NE-CrL1 was associated with a reduction in renal fungal burden, attenuated histopathological alterations, and changes in inflammatory responses in a murine model of invasive candidiasis caused by a fluconazole-resistant *C. albicans* strain. These findings provide preliminary evidence of antifungal activity under physiologically relevant conditions and support the continued evaluation of metal-based coordination complexes in animal models.

Metal coordination complexes have attracted increasing interest as antimicrobial candidates. While their antibacterial properties have been extensively investigated, increasing attention has been directed toward their antifungal activity due to the physicochemical characteristics of metal ions and their potential to interact with biological targets through multiple mechanisms, including redox activity, ligand-triggered release, and multimodal interactions with cellular biomolecules [8,13,17]. Various coordination complexes incorporating metal centers such as Ni(II), Co(II), Cu(II), Cr(III) and Zn(II) have demonstrated antifungal activity [18,19,20,21,22,23,24]. Among them, Cr(III)-based complexes have shown activity against different *Candida* species, including *C. albicans*, *C. tropicalis* and *C. parapsilosis*, with MIC values ranging from 7.81 μg/mL to 125 μg/mL [9]. In addition, Predoi et al. demonstrated that chromium-doped hydroxyapatite nanocomposites inhibit *C. albicans* biofilm formation [25]. In the present study, CrL1 showed higher activity against the fluconazole-resistant CAP F-13 strain compared to the susceptible isolate, suggesting a possible strain-dependent response. Possible explanations include differences in membrane composition or cellular stress responses, as previously described in *Candida* [26,27,28,29]. However, these mechanisms were not investigated in the present study. The free triazole ligand did not exhibit detectable antifungal activity against either strain, even at high concentrations. This observation is consistent with previous reports showing that coordination of ligands to metal centers frequently enhances antimicrobial activity relative to the uncoordinated ligands, which has been associated with increased lipophilicity, improved membrane permeability, enhanced chemical stability, and stronger interactions with biological targets [30]. In addition, the CrL1 complex displayed low cytotoxicity in J774 macrophages within the tested concentration range (CC_50_ > 300 μg/mL), yielding a favorable in vitro selectivity index against the CAP F-13 isolate.

To date, most studies on metal-based coordination complexes remain limited to in vitro evaluations, with relatively few reports addressing their therapeutic potential in animal models of fungal infection [13,31,32]. One of the factors that may contribute to this limitation is the poor aqueous solubility frequently observed in these compounds, which can compromise their bioavailability and pharmacological performance [33]. In this context, nanoemulsion-based systems have been proposed as formulation strategies to improve the dispersion and facilitate the delivery of poorly soluble compounds in biological environments [34,35]. In the present study, the chromium complex CrL1 was incorporated into a nanoemulsion system to enable its in vivo administration. Preliminary physicochemical assessment indicated that the formulation maintained a nanometric size range and physicochemical properties over the evaluated storage period. Nevertheless, further characterization including encapsulation efficiency and drug release profile remains necessary. The Unloaded NE exhibited in vitro antifungal activity only at substantially higher concentrations (MIC = 500 μg/mL), suggesting a limited contribution of the formulation components to the observed effects. Importantly, no direct in vivo comparison between NE-CrL1 and the Unloaded NE was performed, limiting the ability to determine the specific contribution of the formulation to the observed biological effects.

Although studies evaluating metal-based coordination complexes in animal models of fungal infection remain limited, emerging evidence supports their potential in vivo activity. In this context, the reduction in fungal burden and attenuation of tissue alterations observed in the present study are consistent with previous reports. Sovari et al., using a zebrafish model of mixed *C. albicans–MRSA* infection, demonstrated antimicrobial and immunomodulatory effects of a rhenium complex [36]. Similarly, Rubbiani et al. described a ferrocene-bearing fluconazole derivative that reduced fungal burden and improved inflammatory pathology in a murine model of *Candida* infection [37]. Together, these findings support the potential of metal-based compounds as candidates for antifungal evaluation in animal models.

In vivo treatment with NE-CrL1 was associated with a reduction in renal fungal burden in mice infected with the fluconazole-resistant *C. albicans* strain (CAP F-13). In line with these findings, histopathological assessments showed lower necrosis and inflammatory infiltration in treated animals from 24 h post-infection, which may be consistent with attenuation of tissue alterations associated with severe invasive candidiasis [38,39]. Additional observations in brain tissue revealed that NE-CrL1-treated mice showed lower necrosis scores, despite the absence of a clear reduction in fungal burden. Notably, increased neutrophilic infiltration in some groups likely reflects the complex role of neutrophils during invasive candidiasis, which can contribute to both fungal clearance and inflammation-mediated tissue damage [40]. However, these observations should be interpreted cautiously, as histopathological evaluation was performed on a limited number of biological replicates and is presented as a descriptive analysis.

The absence of a significant reduction in brain fungal burden should be considered in this context. The biodistribution of CrL1 and NE-CrL1 was not assessed, and the blood–brain barrier remains a known limitation for the central nervous system exposure of both established and novel antifungal compounds [41]. Further studies are required to evaluate whether the formulation reaches effective concentrations in the brain.

In addition to the effects observed on fungal burden and tissue pathology, NE-CrL1-treated CAP F-13-infected mice showed increased levels of several pro-inflammatory cytokines, including IL-1β, IL-2, IL-12, IL-18 and TNF-α, together with histological evidence of lymphocytic infiltration in renal tissue. These cytokines are known to play key roles in antifungal immunity, contributing to the activation of innate immune responses and the orchestration of adaptive immunity during *Candida* infections. For instance, IL-1β and TNF-α are associated with early inflammatory responses and neutrophil recruitment, whereas IL-12 and IL-18 are involved in the activation of Th1-type responses, which are critical for fungal clearance. In this context, the cytokine profile observed in the present study suggests an immunologically active microenvironment, though sustained elevation of pro-inflammatory cytokines may also contribute to inflammation-mediated tissue damage during invasive candidiasis [40]. Whether this response reflects a protective or a detrimental effect, and whether it directly contributed to infection control, cannot be established from the present data. While metal-containing nanomaterials and related systems have been reported to modulate immune responses [42,43], it remains unclear whether the observed cytokine changes were directly induced by the compound or formulation, or occurred secondary to reduced fungal burden. Therefore, further studies are required to elucidate the mechanisms underlying these immunomodulatory effects.

Several limitations should be considered when interpreting these findings. The antifungal activity was evaluated against a single fluconazole-resistant clinical isolate (CAP F-13), and therefore the observed effects cannot be generalized to other resistant strains. The mechanistic basis of the observed antifungal and immunomodulatory effects was not experimentally investigated. In addition, the physicochemical characterization of the nanoemulsion remains preliminary, as encapsulation efficiency and drug release profiles were not assessed. No direct in vivo comparison between NE-CrL1 and the free CrL1 compound was performed, which limits conclusions regarding the specific contribution of the formulation. Finally, histopathological analyses were based on a limited number of biological replicates and are presented as descriptive observations.

## 5. Conclusions

This study provides preliminary evidence that NE-CrL1 exhibits antifungal activity in a murine model of invasive candidiasis caused by a fluconazole-resistant *C. albicans* strain. Treatment with NE-CrL1 reduced renal fungal burden and was associated with attenuation of histopathological features, together with changes in local inflammatory responses.

These observations support continued evaluation of CrL1 using nanoemulsion-based systems in antifungal studies against drug-resistant *Candida* infections. Nonetheless, clarification of the underlying mechanisms and comprehensive pharmacological characterization, including pharmacokinetics, safety, and dosing optimization, remain necessary before considering any potential translational application.

## Figures and Tables

**Table 1:** Antifungal and cytotoxic activity of chromium(III) complexes, their corresponding triazole ligands, nanoemulsion formulation and reference antifungal drugs.

Compounds	*C. albicans*				Mammalian Cells
	ATCC 90028		CAP F-13		J774A.1
	MIC ^1^	MFC ^2^	MIC ^1^	MFC ^2^	CC_50_ ± SD ^3^
**L1**	>1000	>1000	>1000	>1000	>300
**L2**	>1000	>1000	>1000	>1000	>300
**L3**	>1000	>1000	>1000	>1000	>300
**CrL1**	125	>1000	15.5	500	>300
**CrL2**	>1000	>1000	125	>1000	233.52 ± 13.75
**CrL3**	>1000	>1000	31.25	500	103.32 ± 10.24
**CrCl_3_**·**6H_2_O**	62.25	125	31.25	250	294.44 ± 5.10
**NE** ^4^**-CrL1**	125	>1000	31.25	500	>300
**Unloaded NE**	500	>1000	500	>1000	>300
**Fluconazole**	0.25	>64	>64	>64	>300
**Itraconazole**	0.03	16	0.5	>16	>300

^1^ Minimum inhibitory concentrations (µg/mL). ^2^ Minimum fungicidal concentrations (µg/mL). ^3^ Half-maximal cytotoxic concentrations ± standard deviation (µg/mL). ^4^ Nanoemulsion.

## Data Availability

The original contributions presented in this study are included in the article/Supplementary Material. Further inquiries can be directed to the corresponding authors.

## Supplementary Materials

The following supporting information can be downloaded at https://www.mdpi.com/article/10.3390/jof12060403/s1: Table S1: Colloidal characterization of nanoemulsions; Figure S1: Structural representations of triazole ligands (L1–L3) and their chromium(III) coordination complexes (CrL1–CrL3); Figure S2: Semi-quantitative histopathological scoring of brain lesions in mice infected with *C. albicans*.

## Abbreviations

The following abbreviations are used in this manuscript:

ATCC	American Type Culture Collection
BCA	Bicinchoninic Acid
CC_50_	Half-maximal Cytotoxic Concentration
CFU	Colony-Forming Unit
CLSI	Clinical and Laboratory Standards Institute
CrL1, CrL2, CrL3	Chromium(III) coordination complexes with triazole ligands 1, 2, 3
CrCl_3_·6H_2_O	Chromium(III) chloride hexahydrate
*D*_h_	Hydrodynamic Diameter
DLS	Dynamic Light Scattering
DMEM	Dulbecco’s Modified Eagle Medium
DMSO	Dimethyl Sulfoxide
ELS	Electrophoretic Light Scattering
FLZ	Fluconazole
GM-CSF	Granulocyte Macrophage Colony Stimulating Factor
H&E	Hematoxylin and Eosin
IC	Invasive Candidiasis
IFI	Invasive Fungal Infection
IFN-γ	Interferon Gamma
IL	Interleukin
MFC	Minimal Fungicidal Concentration
MIC	Minimal Inhibitory Concentration
NE	Nanoemulsion
NE-CrL1	CrL1-loaded Nanoemulsion
PBS	Phosphate-Buffered Saline
PDI	Polydispersity Index
ROS	Reactive Oxygen Species
RPMI	Roswell Park Memorial Institute medium
SD	Standard Deviation
SI	Selectivity Index
TNF-α	Tumor Necrosis Factor Alpha
Unloaded NE	CrL1-free Nanoemulsion
ZP	Zeta Potential
