# Supplementary material for: In Vivo Evaluation of a Nanoemulsion-Delivered Chromium(III)–Triazole Complex Against Fluconazole-Resistant Candida albicans
Source: J Fungi (Basel). 2026 Jun 2;12(6):403. doi: 10.3390/jof12060403 (PMC13301959; doi:10.3390/jof12060403)
Supplement: Supplementary file 1 [file jof-12-00403-s001.zip › jof-4308615-supplementary.pdf]

Table S1. Colloidal characterization of nanoemulsions

Nanoemulsion	Months	D_h[1]	PDI[2]	ZP[3]
NE-CrL1	1	82.90±1.40	0.461±0.00	1.18±0.07
NE-CrL1	4	85.59±1.18	0.464±0.00	1.33±0.04
Unloaded NE	1	73.30±2.39	0.551±0.02	-2.53±0.21
Unloaded NE	4	78.07±0.9	0.528±0.00	-2.86±0.27

[1] Hydrodynamic diameter (nm).

[2] Polydispersity index (unitless).

[3] Zeta potential (mV).

[4]Data are expressed as means ± standard deviation (SD) from at least two independent experiments.

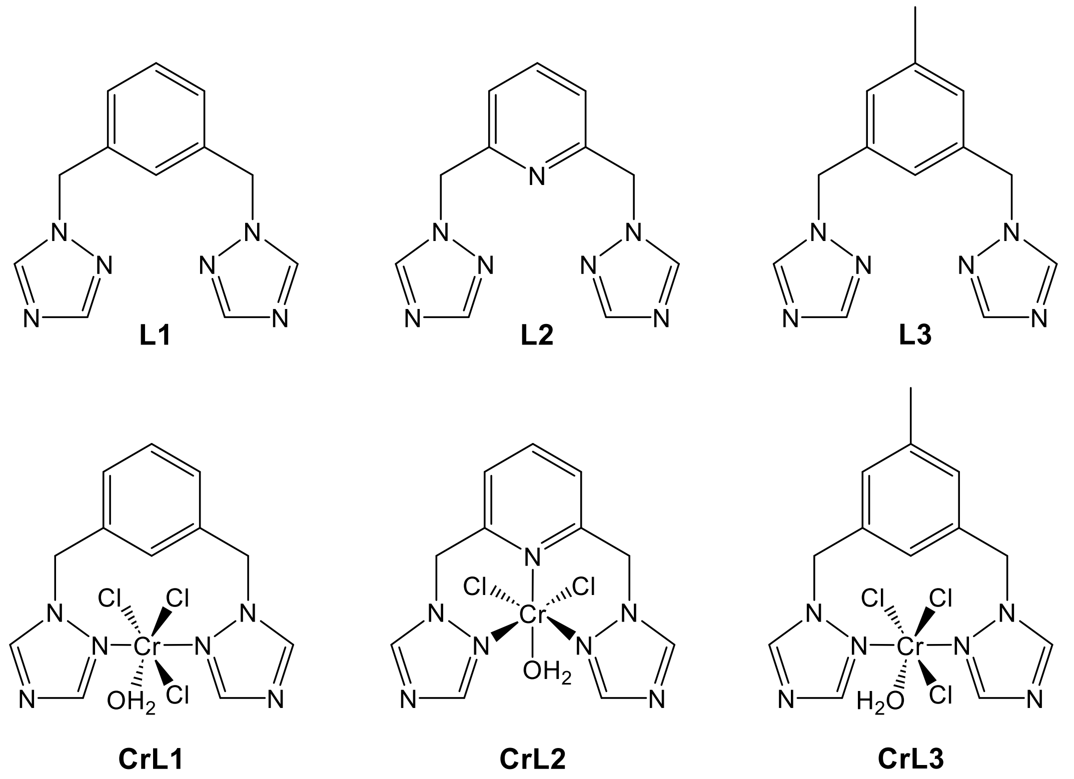

Figure S1. Structural representations of triazole ligands (L1–L3) and their chromium(III) coordination complexes (CrL1–CrL3).

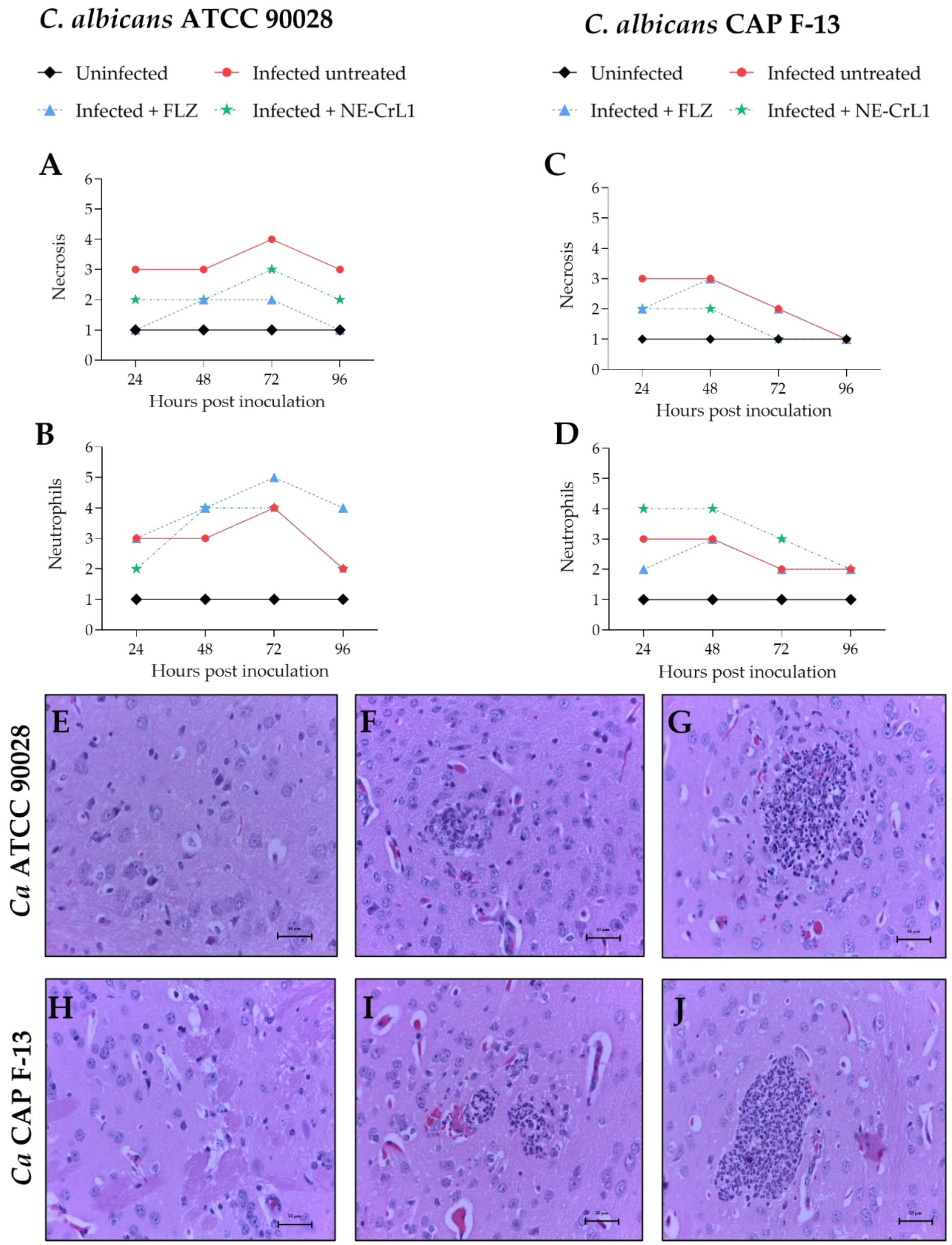

Figure S2. Semi-quantitative histopathological scoring of brain lesions in mice infected with *C. albicans*. Brain tissue from mice infected with fluconazole-susceptible (ATCC 90028) and fluconazole-resistant (CAP F-13) was evaluated for (A,C) necrosis and (B,D) neutrophil infiltration. Each parameter was graded on a severity scale from 1 (no lesion) to 6 (severe lesion). (E-J) Representative microphotographs of H&E staining brain tissue of infected untreated (E,H), Infected + FLZ (F,I) and Infected + CrL1 (G,J) at 96 hours post infection. Magnification at 40X.
